# ﻿*Exophialayunnanensis* and *Exophialayuxiensis* (Chaetothyriales, Herpotrichiellaceae), two new species of soil-inhabiting *Exophiala* from Yunnan Province, China

**DOI:** 10.3897/mycokeys.94.96782

**Published:** 2022-12-21

**Authors:** Ruili Lv, Xiaoqian Yang, Min Qiao, Linlin Fang, Jianying Li, Zefen Yu

**Affiliations:** 1 Laboratory for Conservation and Utilization of Bio-resources, Key Laboratory for Microbial Resources of the Ministry of Education, Yunnan University, Kunming, Yunnan, 650091, China Yunnan University Kunming China; 2 Kunming Edible Fungi Institute of All China Federation of Supply and Marketing Cooperatives, Kunming 650221, China Kunming Edible Fungi Institute of All China Federation of Supply and Marketing Cooperatives Kunming China

**Keywords:** *Exophiala*, multi-locus phylogeny, morphology, new species, taxonomy

## Abstract

During a survey of soil fungi collected from Yunnan Province, China, two new species of *Exophiala*, *E.yunnanensis* and *E.yuxiensis*, were isolated from the soil of karst rocky desertification (KRD). The DNA sequences of these respective strains, including internal transcribed spacers (ITS), large subunit nuclear ribosomal RNA (LSU rRNA), partial small subunit (SSU) and β-tubulin (*tub2*) were sequenced and compared with those from species closely-related to *Exophiala*. *Exophialayunnanensis* differs from the phylogenetically closely related *E.nagquensis* and *E.brunnea* by its smaller aseptate conidia. *Exophialayuxiensis* is phylogenetically related to *E.lecanii-corni*, *E.lavatrina* and *E.mali*, but can be distinguished from them by its larger conidia. Full descriptions, illustrations and phylogenetic positions of *E.yunnanensis* and *E.yuxiensis* were provided.

## ﻿Introduction

*Exophiala* J.W. Carmich. (Chaetothyriales, Herpotrichiellaceae) was established with *E.salmonis* J.W. Carmich. as type species (Carmichael 1966) in Alberta, Canada. Due to their yeast-like melanised colonies, these fungi are often also referred to as “black yeasts” (Matsumoto et al. 1987). The genus is characterised by annellidic conidiogenous cells producing slimy heads of conidia, conidiophores upright or bent, not or irregularly branched, smooth, light olive to brown. However, there are several synanamorphs recorded in this genus (Thitla et al. 2022). Nearly all species are recognisable within the order by the way they produce cells by budding (De Hoog et al. 2011).

*Exophiala* spp. are widely distributed and can be isolated from bulk soil, biological crusts, rock surfaces, air, natural water masses, rhizosphere, plant tissues, and infected animals and human tissue (Addy et al. 2005; Bates et al. 2006; Neubert et al. 2006; Bukovská et al. 2010; Julou et al. 2010; De Hoog et al. 2011). Most studies on *Exophiala* species focused on their importance as etiologic agents of disease in animals and humans (Zeng and De Hoog 2008; Najafzadeh et al. 2013; Wen et al. 2016). Several *Exophiala* species are opportunistic pathogens of immunocompetent humans (Woo et al. 2013; Yong et al. 2015), in rare occasions causing nervous system phaeohyphomycosis (Chang et al. 2000) or causing cutaneous and subcutaneous skin infections, including *E.spinifera* (H.S. Nielsen & Conant) Mcginnis, which has the strongest pathogenicity to human skin (Vitale and De Hoog 2002). Furthermore, some *Exophiala* species, such as *E.salmonis*, *E.aquamarina* de Hoog et al. and *E.equina* (Pollacci) de Hoog et al. may cause cutaneous or disseminated infections of cold-blooded animals (De Hoog et al. 2011). Therefore, the classification and identification of this genus are significantly important for clinical diagnosis, treatment and prevention.

In the past, taxonomic and diagnostic schemes for *Exophiala* were morphological characteristics, but the anamorphic states of some species are highly pleomorphic (De Hoog et al. 1995; Haase et al. 1995; Thitla et al. 2022), which make them difficult to be recognised and circumscribed (Naveau 1999; Zeng and De Hoog 2008), so only a small number of *Exophiala* species are, in fact, recognisable using morphology. With the development of molecular systematics, more and more species were redefined, re-designated or described mainly depending on genetic, morphological, physiological and ecological features (Haase et al. 1999; De Hoog et al. 2003; Vitale et al. 2003; De Hoog et al. 2006). At present, 80 names in *Exophiala* were recorded in Index Fungorum, amongst them *E.nigra* (Issatsch.) Haase & de Hoog, *E.placitae* Crous & Summerell, *E.prototropha* (Bulanov & Malama) Haase et al. and *E.werneckii* (Horta) Arx, have been moved to *Nadsoniella* Issatsch., *Neophaeococcomyces* Crous & M.J. Wingf., *Pullularia* Berkhout and *Hortaea* Nishim. & Miyaji, respectively. Currently, 68 species have been accepted into this genus after a brief review of Thitla et al. (2022) and Crous et al. (2022), who described new species from Thailand and Australia.

During a survey of fungi from rocky desertification area, two unknown fungi were found. Based on morphology and phylogenetic analysis combined ITS, SSU, LSU and *tub2*, we proposed two new species, *E.yunnanensis* and *E.yuxiensis*.

## ﻿Materials and methods

### ﻿Isolation and morphological characterisation of strains

Soil samples were collected from Yiliang and Yuxi in Yunnan Province, southwest China. Samples were placed in plastic bags, labelled and transported to the laboratory. All the samples were stored at 4 °C before further processing. Fungal strains were obtained by serial dilutions (1,000 to 1,000,000 fold) and spread on to the surface of Rose Bengal agar with antibiotics (40 mg streptomycin, 30 mg ampicillin per litre) added in a 9 cm diam. Petri dish, followed by incubation at 25 °C for 5 days (Zheng et al. 2021a). Representative colonies were picked up with a sterilised needle and transferred to potato dextrose agar (PDA, 200 g potato, 20 g dextrose, 18 g agar, 1000 ml distilled water). After 7 days, colonies were transferred to cornmeal agar (CMA, 20 g cornmeal, 18 g agar, 1000 ml distilled water). Characteristics of colonies, growth rate and other morphological aspects from PDA were observed after 10 days. Microscopic characteristics including mycelium, 10 conidiophores and 30 conidia were examined and measured after 7 days on CMA using an Olympus BX51 microscope. Pure cultures were deposited in the Herbarium of the Laboratory for Conservation and Utilization of Bio-Resources, Yunnan University, Kunming, Yunnan, P.R. China (**YMF**, formerly Key Laboratory of Industrial Microbiology and Fermentation Technology of Yunnan), China General Microbiological Culture Collection Center (**CGMCC**), the Guangdong Microbial Culture Collection Center (**GDMCC**) and Japan Collection of Microorganisms (**JCM**).

### ﻿DNA extraction, PCR amplification and sequencing

Total DNA was extracted following the protocol of Zheng et al. (2021b). The internal transcribed spacer (ITS), the large subunit nuclear ribosomal RNA (LSU rRNA), the partial small subunit (SSU) and the β-tubulin (*tub2*) were amplified using the primer pairs ITS1/ITS4 (White 1990), LR0R/LR5 (Vilgalys and Hester 1990), NSSU131/NS24 (Kauff and Lutzoni 2002) and Bt2a/Bt2b (Glass and Donaldson 1995), respectively. The PCR amplifications were conducted in 25 µl final volumes which consisted of 1.0 µl DNA template, 1.0 µl of each forward and reverse primers, 12.5 µl 2 × Master Mix and 9.5 µl ddH_2_O. The PCR reaction cycles were as follows: initial denaturation at 94 °C for 5 min; followed by 35 cycles of denaturation at 94 °C for 40 s; the annealing extension dependent on the amplified loci (48 °C for LSU, 54 °C for ITS, 51 °C for SSU and 58 °C for *tub2*) for 1 min and extension at 72 °C for 2 min; a final extension at 72 °C for 10 min. PCR products were sequenced by TSINGKE Biological Technology in Kunming, China.

### ﻿Sequence alignment and phylogenetic analysis

Preliminary BLAST searches with ITS, LSU, SSU and *tub2* gene sequences of the new isolates against NCBI databases had identified species closely related to our two isolates. Based on this information, ITS, LSU, SSU and *tub2* sequences of 62 strains were downloaded and used in the phylogenetic analysis with *Cyphellophoraoxyspora* (CBS 698.73) as outgroup. The GenBank accession numbers of sequences used in the phylogenetic analysis are shown in Table 1. DNA sequence data were aligned using ClustalX 1.83 (Thompson et al. 1997) with default parameters. Aligned sequences of multiple loci were concatenated and manually adjusted through BioEdit version v. 7.0.4.1 (Hall 1999) and ambiguously aligned regions were excluded. The combined sequence was converted to a NEXUS file using MEGA6 (Tamura et al. 2013) and it was uploaded to TreeBASE (www.treebase.org; accession number: S29757).

**Table 1.** Species, strains and their corresponding GenBank accession numbers of sequences used for phylogenetic analyses. *Exophiala* strains of the present study were marked in bold. ^T^ex-type cultures; “-” The gene fragment representing this strain was not attainable.

Species	Strain no.	GenBank accession no.			
		ITS	LSU	SSU	*tub*2
*Exophialaabietophila*	CBS 145038^T^	MK442581	NG066323	–	–
*Exophialaalcalophila*	CBS 520.82^T^	JF747041	AF361051	JN856010	JN112423
*Exophialaangulospora*	CBS 482.92^T^	JF747046	KF155190	JN856011	JN112426
*Exophialaaquamarina*	CBS 119918^T^	JF747054	–	JN856012	JN112434
*Exophialaasiatica*	CBS 122847^T^	EU910265	–	–	–
*Exophialaattenuata*	F10685	KT013095	KT013094	–	–
*Exophialabergeri*	CBS 353.52^T^	EF551462	FJ358240	FJ358308	EF551497
*Exophialabonariae*	CBS 139957^T^	JX681046	KR781083	–	–
*Exophialabrunnea*	CBS 587.66^T^	JF747062	KX712342	JN856013	JN112442
*Exophialacampbellii*	NCPF 2274	LT594703	LT594760	–	–
*Exophialacancerae*	CBS 120420^T^	JF747064	–	–	JN112444
*Exophialacapensis*	CBS 128771^T^	JF499841	MH876538	–	–
*Exophialacastellanii*	CBS 158.58^T^	JF747070	KF928522	JN856014	KF928586
*Exophialacinerea*	CGMCC 3.18778^T^	MG012695	MG197820	MG012724	MG012745
*Exophialaclavispora*	CGMCC 3.17512	KP347940	MG197829	MG012733	KP347931
*Exophialacrusticola*	CBS 119970^T^	AM048755	KF155180	KF155199	–
*Exophialadermatitidis*	CBS 207.35^T^	AF050269	KJ930160	–	KF928572
*Exophialaellipsoidea*	CGMCC 3.17348^T^	KP347955	KP347956	KP347965	KP347921
*Exophialaembothrii*	CBS 146560	MW045819	MW045823	–	–
*Exophialaequina*	CBS 119.23^T^	JF747094	–	JN856017	JN112462
*Exophialaeucalypti*	CBS 142069	KY173411	KY173502	–	–
*Exophialaeucalyptorum*	CBS 121638^T^	NR132882	KC455258	KC455302	KC455228
*Exophialaexophialae*	CBS 668.76^T^	AY156973	KX822326	KX822287	EF551499
*Exophialafrigidotolerans*	CBS 146539^T^	LR699566	LR699567	–	–
*Exophialahalophila*	CBS 121512^T^	JF747108	–	JN856015	JN112473
*Exophialaheteromorpha*	CBS 232.33^T^	MH855419	MH866871	–	–
*Exophialahongkongensis*	CBS 131511	JN625231	–	–	JN625236
*Exophialaitalica*	MFLUCC 16-0245^T^	KY496744	KY496723	KY501114	–
*Exophialajeanselmei*	CBS 507.90^T^	AY156963	FJ358242	FJ358310	EF551501
*Exophialalacus*	FMR 3995	KU705830	KU705847	–	–
*Exophialalavatrina*	NCPF 7893	LT594696	LT594755	–	–
*Exophialalecanii-corni*	CBS 123.33^T^	AY857528	FJ358243	FJ358311	–
*Exophialalignicola*	CBS 144622^T^	MK442582	MK442524	–	–
*Exophialamacquariensis*	CBS 144232^T^	MF619956	–	–	MH297438
*Exophialamali*	CBS 146791^T^	MW175341	MW175381	–	–
*Exophialamansonii*	CBS 101.67^T^	AF050247	AY004338	X79318	–
*Exophialamesophila*	CBS 402.95^T^	JF747111	KX712349	JN856016	JN112476
*Exophialamoniliae*	CBS 520.76^T^	KF881967	KJ930162	–	–
*Exophialanagquensis*	CGMCC 3.17284	KP347947	MG197838	MG012742	KP347922
*Exophialanidicola*	FMR 3889	MG701055	MG701056	–	–
*Exophialanigra*	CBS 535.94^T^	KY115191	KX712353	–	–
*Exophialanishimurae*	CBS 101538^T^	AY163560	KX822327	KX822288	JX482552
*Exophialaoligosperma*	CBS 725.88^T^	AY163551	KF928486	FJ358313	EF551508
*Exophialaopportunistica*	CBS 109811^T^	JF747123	KF928501	–	JN112486
*Exophialapalmae*	CMRP 1196^T^	KY680434	KY570929	–	KY689829
*Exophialaphaeomuriformis*	CBS 131.88^T^	AJ244259	–	–	–
*Exophialapisciphila*	CBS 537.73^T^	NR121269	AF361052	JN856018	JN112493
*Exophialaplacitae*	CBS 121716^T^	MH863143	MH874694	–	–
*Exophialaprostantherae*	CBS 146794^T^	MW175344	MW175384	–	–
*Exophialapolymorpha*	CBS 138920^T^	KP070763	KP070764	–	–
*Exophialapseudooligosperma*	YMF 1.6741	MW616557	MW616559	MW616558	MZ127830
*Exophialapsychrophila*	CBS 191.87^T^	JF747135	–	JN856019	JN112497
*Exophialaquercina*	CPC 33408	MT223797	MT223892	–	–
*Exophialaradicis*	P2772	KT099203	KT723447	KT723452	KT723462
*Exophialasalmonis*	CBS 157.67^T^	AF050274	AY213702	JN856020	JN112499
*Exophialasideris*	CBS 121818^T^	HQ452311	–	HQ441174	HQ535833
*Exophialaspinifera*	CBS 899.68^T^	AY156976	–	–	EF551516
*Exophialatremulae*	CBS 129355^T^	FJ665274	–	KT894147	KT894148
*Exophialaxenobiotica*	CBS 128104	MH864829	MH876272	–	–
***Exophialayunnanensis***	**YMF1.06739**	**MZ779226**	**MZ779229**	**MZ781222**	**OM095379**
***Exephialayuxiensis***	**YMF1.07354**	**OL863155**	**OL863154**	**OM149370**	**OL944581**
*Cyphellophoraoxyspora*	CBS 698.73^T^	KC455249	KC455262	KC455305	KC455232

Phylogenetic analyses were conducted using both the Bayesian Inference (BI) and Maximum Likelihood (ML) methods. Bayesian Inference analysis was conducted using MrBayes v.3.2 (Ronquist et al. 2012) with the NEXUS file. The following parameters were used: ngen = 1,000,000; samplefr = 1,000; printfr = 1,000. The Akaike Information Criterion (AIC) implemented in jModelTest version 2.0 (Posada 2008) was used to select the best fit models after likelihood score calculations were done. TPM1uf + I + G was estimated as the best-fit model under the output strategy of AIC. Two independent analyses with four chains each (one cold and three heated) were run until stationary distribution was achieved. The initial 25% of the generations of MCMC sampling were excluded as burn-in. The refinement of the phylogenetic tree was used for estimating Bayesian Inference posterior probability (BIPP) values. The ML trees, based on four gene loci, were constructed with the GTR+GAMMA model using RAxML version 7.2.6 (Stamatakis 2006) and the robustness of branches was assessed by bootstrap analysis with 1000 replicates. The tree was viewed in TreeView 1.6.6 (Page 1996) with Maximum Likelihood bootstrap proportions (MLBP) greater than 50% and Bayesian Inference posterior probabilities (BIPP) greater than 70%, as shown at the nodes.

## ﻿Results

### ﻿Molecular phylogeny

**Figure 1.**
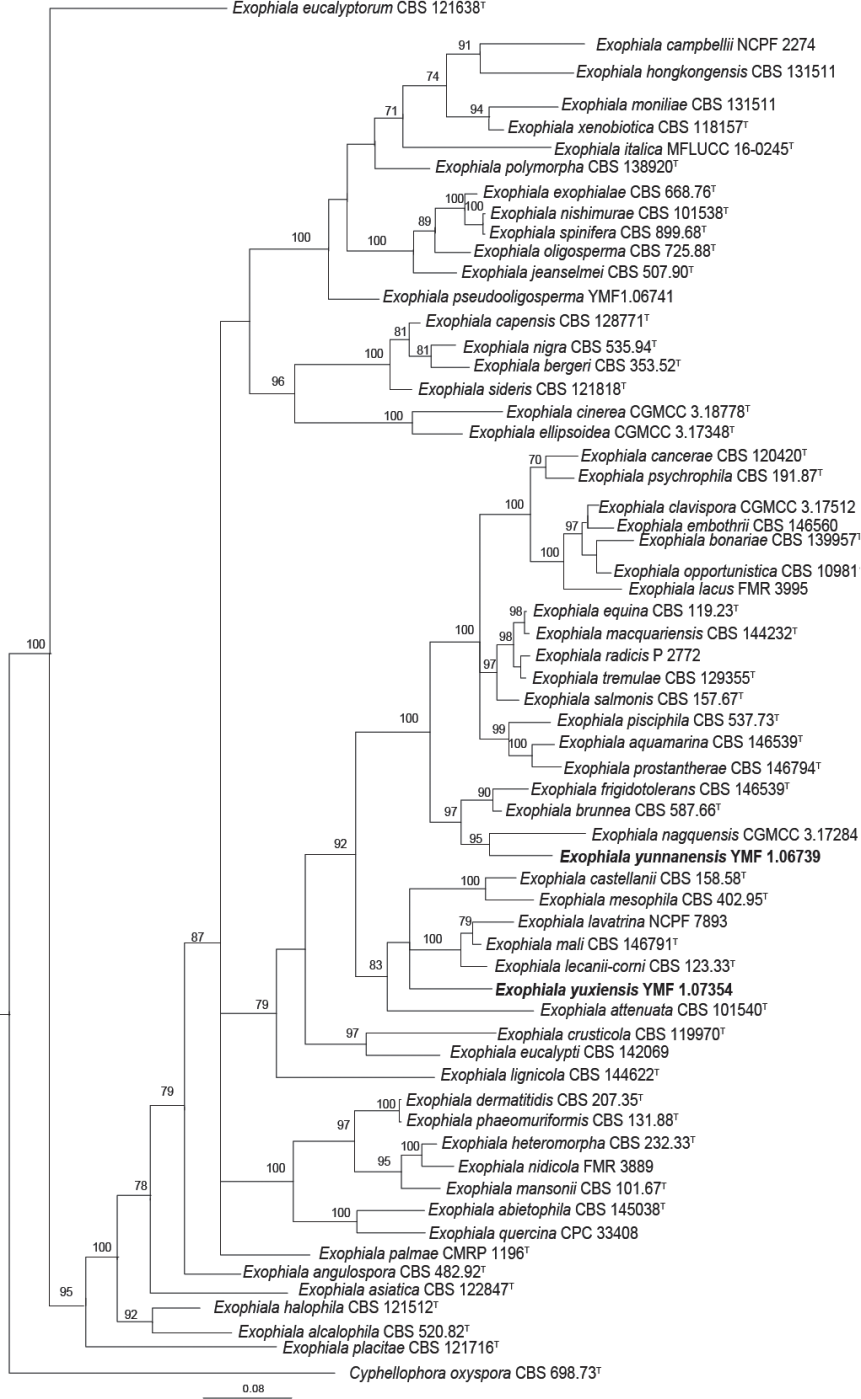
Phylogenetic tree generated by Bayesian Inference, based on sequences of the ITS. *Cyphellophoraoxyspora* CBS 698.73 serves as outgroup. Bayesian posterior probability over 75 is shown at the nodes. Two new species were shown in bold.

The Bayesian tree, based on ITS sequence data, confirmed that two strains were distinct from known species of *Exophiala* (Fig. 1), *Exophialayunnanensis* is phylogenetically close to *E.nagquensis*CGMCC 3.17284 and ITS similarity between *E.yunnanensis* and *E.nagquensis* is 92.21%. *Exophialayuxiensis* is phylogenetically related to *E.lecanii-corni* CBS 123.33, *E.mali* CBS 146791 and *E.lavatrina* NCPF 7893 and the similarities between the holotype of *E.yuxiensis* and the representative strains of three species are 90.27%, 89.86% and 85.08%, respectively.

**Figure 2.**
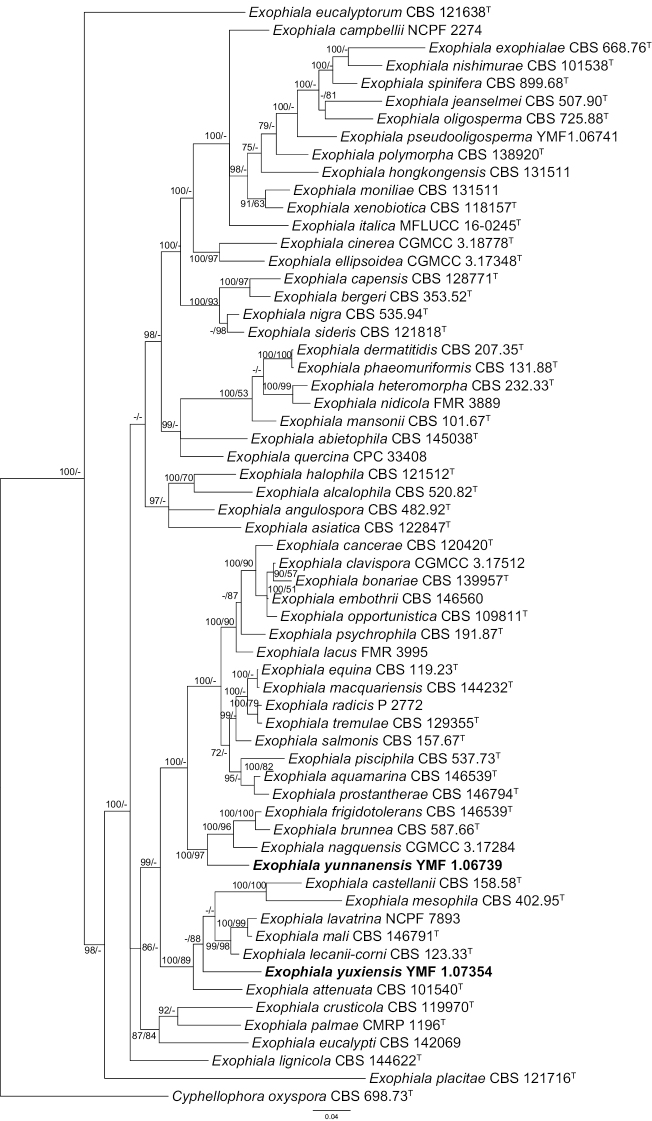
Phylogenetic tree generated by Bayesian analyses combined sequences of ITS, LSU, SSU and *tub2*. Bayesian posterior probability values > 70 (left) and Bootstrap values > 50 (right) are indicated at nodes (BIBP/MLBP). *Cyphellophoraoxyspora* CBS 698.73 serves as outgroup.

In the combined phylogenetic analyses (ITS, LSU, SSU and *tub2*), which contained 2218 characters, a similar topological structure was observed between the two phylogenetic trees constructed by BI and ML. The support values with BI analysis are relatively higher than the ML bootstrap support values (Fig. 2) In this tree, *E.yunnanensis*, *E.nagquensis* W. Sun et al., *E.brunnea* Papendorf and *E.frigidotolerans* Rodr.-Andr. et al. formed a clade with high statistical support (BIBP/MLBP = 100/97). *Exophialayuxiensis* is phylogenetically close to *E.lecanii-corni* (Benedek & G. Specht) Haase & de Hoog and the clade formed by these species and six additional ones also has high statistical support (BIBP/MLBP = 100/89).

### ﻿Taxonomy

#### ﻿ Exophiala yunnanensis

Taxon classificationFungiChaetothyrialesHerpotrichiellaceae

Z.F. Yu & R.L. Lv sp. nov.

89BB45D0-14C4-5677-87C0-7CA85E538C2E

 842373

Fig. 3

##### Etymology.

*yunnanensis*, pertaining to Yunnan, a province of southwest China from where the type was collected.

##### Description.

Colonies on CMA medium after 7 days with hyphae olive green, smooth, septate, thin walled, branched, 1.6–3.0 µm wide. Conidiogenous cells slightly differentiated from simple or branched vegetative hyphae, terminal or intercalary, flask-shaped, ovoid to elongate, pale brown, loci at tips and lateral; annellated zones inconspicuous or occasionally finely fimbriate, often inserted on intercalary cells. Conidia aseptate, ellipsoidal, cylindrical or allantoid, 1–2 guttulate, smooth, brown, 2.9–4.8 × 1.8–3.3 µm, with a conspicuous scar of approx. 1 µm wide at the base, containing no evident or few small oil drops.

**Figure 3.**
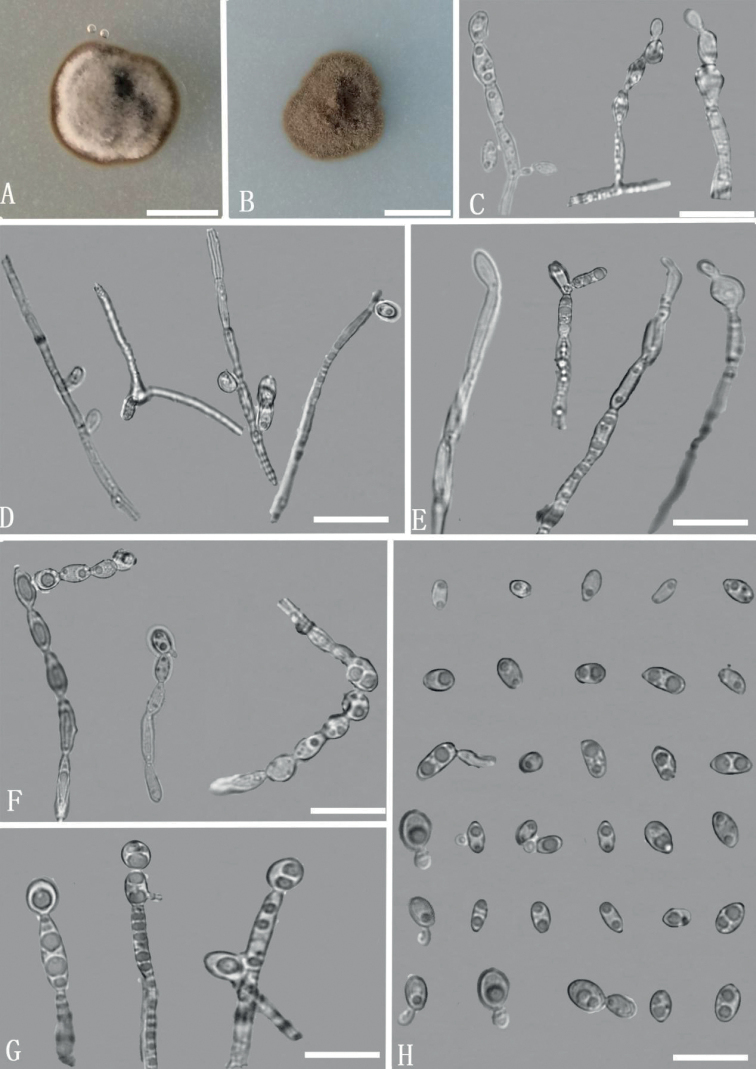
*Exophialayunnanensis* (YMFT 1.06739, holotype) **A** colony on PDA after 14 days **B** colony on CMA after 14 days **C–G** conidiogenous cells **H** conidia and budding cells. Scale bars: 3.2 cm (**A, B**); 10 µm (**C–H**).

##### Culture characteristics.

Colonies on PDA medium, at 25 °C, were slow-growing, mycelium immersed and partly superficial, irregular, umbonate, surface olivaceous-grey to black. Radial growth rates were 0.8–0.9 mm day^-1^on PDA. Colonies on CMA medium were restricted, mycelium immersed and partly superficial, effuse, cottony, reverse olivaceous-buff to olivaceous, reaching 12 mm diam. in 15 days at 25 °C.

##### Type.

**China.** Yiliang County, Yunnan Province, isolated from soil of rocky desertification area, 24°96'N, 102°66'E, ca. 1886 m elev., Oct 2020, Z.F.Yu, preserved by lyophilisation (a metabolically-inactive state) in State Key Laboratory for Conservation and Utilization of Bio-Resources in Yunnan (holotype YMFT 1.06739), ex-holotype live culture: YMF 1.06739; CGMCC 3.16095; GDMCC 3.725; JCM 39339.

#### ﻿ Exophiala yuxiensis

Taxon classificationFungiChaetothyrialesHerpotrichiellaceae

Z.F. Yu & R.L. Lv sp. nov.

E45A7AA1-B151-52AC-AF3F-32056AD63765

 MB842374

Fig. 4

##### Etymology.

*yuxiensis*, pertaining to Yuxi, a city of Yunnan Province in China, from which the type was collected.

##### Description.

Colonies on CMA medium after 7 days with hyphae pale olivaceous-green, smooth, irregularly septate, thin-walled, branched, 1.5–3 µm wide, with lateral branches originating close to septa. Conidiogenous cells slightly differentiated from hyphae, arising from hyphal tips or lateral, terminal or intercalary, variable in shape, flask-shaped, ovoid to elongate, clavate, obtuse at the base, tapering towards inconspicuous annellate loci, 5.5–10.5 × 3–5 μm; annellated zones inconspicuous or occasionally finely fimbriate, often inserted on intercalary cells of hyphae. Conidia aseptate, ellipsoidal to cylindrical, 1–2 (mostly 2) bi-guttulate, smooth, pale olivaceous-green, 4.5–8 × 3.5–5 µm, without conspicuous scar.

**Figure 4.**
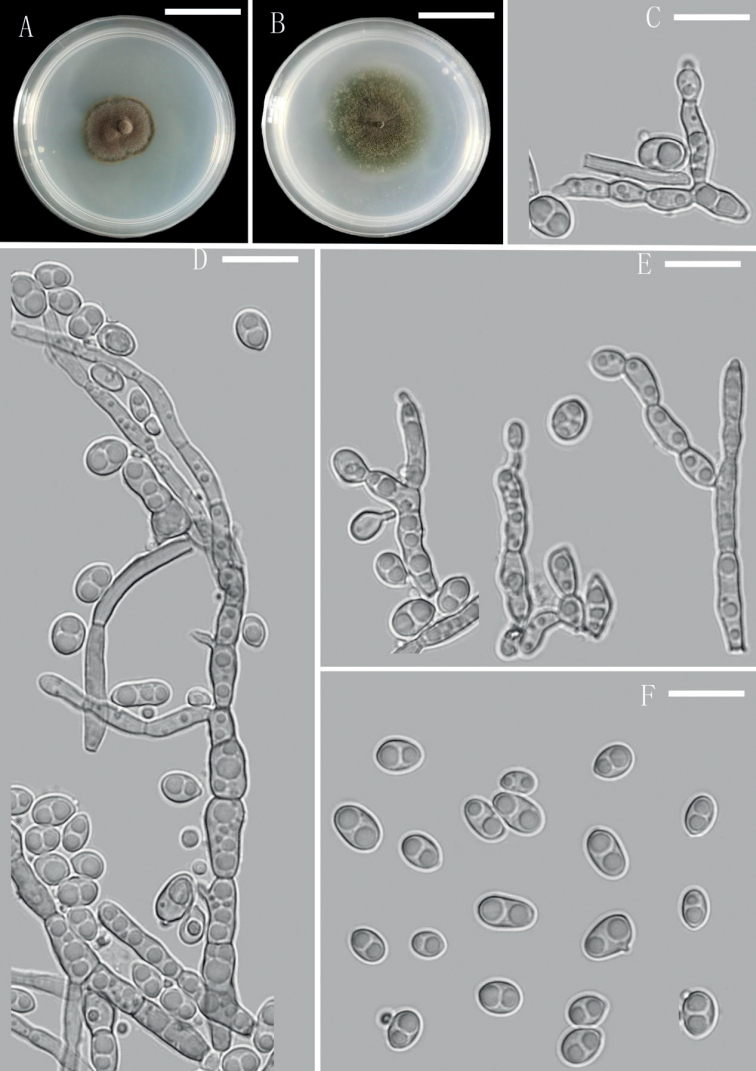
*Exophialayuxiensis* (YMFT 1.07354, holotype) **A** colony on PDA after 30 days **B** colony on CMA after 30 days **C–E** conidiogenous cells **F** conidia and budding cells. Scale bars: 3.2 cm (**A, B**), 10 µm (**C–F**).

##### Culture characteristics.

Colonies on PDA medium, at 25 °C, were slow-growing, mycelium immersed and partly superficial, umbonate, dense, powdery or velvety, dry, margin irregular, surface olivaceous-grey, reverse olivaceous-black, attaining 1 cm diam. in 4 days. Colonies on CMA medium were restricted, mycelium immersed and partly superficial, cottony, surface olivaceous-green, some floccose aerial hyphae in the centre, front distinct, reverse pale olivaceous-black, reaching 3 cm diam. in 5–7 days.

##### Type.

**China.** Yuxi City, Yunnan Province, isolated from soil of rocky desertification area, 24°44'N, 102°55'E, 1660 m altitude, Jul 2021, Z.F. Yu, preserved by lyophilisation (a metabolically-inactive state) in State Key Laboratory for Conservation and Utilization of Bio-Resources in Yunnan (holotype YMFT 1.07354), ex-holotype live culture: YMF 1.07354; CGMCC 3.16094; GDMCC 3.726; JCM 39376).

## ﻿Discussion

In this study, we propose two new species of *Exophiala*, based on combined morphological characteristics and phylogenetic analyses. *Exophialayunnanensis* and *E.yuxiensis* are different from their phylogenetically closely-related species. Amongst them, *E.nagquensis* (Sun et al. 2020) and *E.brunnea* (Papendorf 1969) are distinguished from *E.yunnanensis* by their larger conidia (*E.nagquensis*: 4.8–10.4 × 2.6–5.0 µm; *E.brunnea*: 4.5–10 µm in length; *E.yunnanensis* 2.9–4.8 × 1.8–3.3 µm), while *E.frigidotolerans* differs from *E.yunnanensis* by ellipsoidal to reniform and larger conidia (4.0–7.0 × 2.0–4 .0 µm) (Crous et al. 2020). Additionally, *E.yunnanensis* resembles *E.nagquensis* and *E.frigidotolerans* in the shape of budding cells, but *E.yunnanensis* has smaller budding cells (Maciá-Vicente et al. 2016; Sun et al. 2020).

*Exophialayuxiensis* is phylogenetically related to *E.lecanii-corni*, *E.lavatrina* Borman et al. and *E.mali* Crous. Amongst these species, *E.mali* is the most similar to *E.yuxiensis* by ellipsoidal to cylindrical conidia, but the conidia of *E.mali* are larger (8.0–10.0 × 3.0–5.0 µm vs. 4.5–8.0 × 3.5–5.0 µm) and the hyphae of *E.mali* are constricted at the septa in the terminal region, forming chains of disarticulating conidia (Crous et al. 2020). *Exophialalavatrina* can be distinguished from *E.yuxiensis* by smaller conidia (4.5–7 × 2.5–4 µm) (Borman et al. 2017).

The species of *Exophiala* have a wide distribution, with isolation from diverse substrates, such as plants, fruit juices, shower rooms, seawater, sports drinks, arable soil, wood pulp, oil sludge and the decaying shell of babassu coconut (De Hoog et al. 1994; De Hoog et al. 2006; De Hoog et al. 2011; Feng et al. 2014; Madrid et al. 2016). Some species were reported as opportunistic pathogens on the superficial skin or internal organs in humans and animals. For example, the type species *E.salmonis*, was isolated from cerebral mycetoma of *Salmoclarkii* Richardson, 1836 (Carmichael 1966), while isolates of *E.equina* (Pollacci) de Hoog et al. and *E.pisciphila* McGinnis & Ajello cause disease on cold-blooded animals such as fish, turtles, crabs, sea horses and frogs (De Hoog et al. 2011). In addition, some species were frequently isolated as endophytes (Addy et al. 2005), although they seldom represent important components of endophytic communities.

The present work increased the number of *Exophiala* species to 70 in the world (Crous et al. 2022; Thitla et al. 2022). In China, Yunnan Province has diverse climate and vegetation, which provides natural advantages for the study of environmental microbial diversity. However, further extensive samplings and investigation of fungi are necessary to generate a complete knowledge about the biodiversity, distribution, habitats and adaptation mechanisms from *Exophiala* to environmental stresses.

## Supplementary Material

XML Treatment for Exophiala yunnanensis

XML Treatment for Exophiala yuxiensis
